# Lamin A/C Haploinsufficiency Modulates the Differentiation Potential of Mouse Embryonic Stem Cells

**DOI:** 10.1371/journal.pone.0057891

**Published:** 2013-02-25

**Authors:** Poonam Sehgal, Pankaj Chaturvedi, R. Ileng Kumaran, Satish Kumar, Veena K. Parnaik

**Affiliations:** CSIR-Centre for Cellular and Molecular Biology, Hyderabad, India; University of Newcastle upon Tyne, United Kingdom

## Abstract

**Background:**

Lamins are structural proteins that are the major determinants of nuclear architecture and play important roles in various nuclear functions including gene regulation and cell differentiation. Mutations in the human lamin A gene cause a spectrum of genetic diseases that affect specific tissues. Most available mouse models for laminopathies recapitulate disease symptoms for muscle diseases and progerias. However, loss of human lamin A/C also has highly deleterious effects on fetal development. Hence it is important to understand the impact of lamin A/C expression levels on embryonic differentiation pathways.

**Methodology and Principal Findings:**

We have investigated the differentiation potential of mouse embryonic stem cells containing reduced levels of lamin A/C by detailed lineage analysis of embryoid bodies derived from these cells by *in vitro* culture. We initially carried out a targeted disruption of one allele of the mouse lamin A/C gene (*Lmna*). Undifferentiated wild-type and *Lmna^+/−^* embryonic stem cells showed similar expression of pluripotency markers and cell cycle profiles. Upon spontaneous differentiation into embryoid bodies, markers for visceral endoderm such as α-fetoprotein were highly upregulated in haploinsufficient cells. However, neuronal markers such as β-III tubulin and nestin were downregulated. Furthermore, we observed a reduction in the commitment of *Lmna^+/−^* cells into the myogenic lineage, but no discernible effects on cardiac, adipocyte or osteocyte lineages. In the next series of experiments, we derived embryonic stem cell clones expressing lamin A/C short hairpin RNA and examined their differentiation potential. These cells expressed pluripotency markers and, upon differentiation, the expression of lineage-specific markers was altered as observed with *Lmna^+/−^* embryonic stem cells.

**Conclusions:**

We have observed significant effects on embryonic stem cell differentiation to visceral endoderm, neuronal and myogenic lineages upon depletion of lamin A/C. Hence our results implicate lamin A/C level as an important determinant of lineage-specific differentiation during embryonic development.

## Introduction

The nuclear lamins are type V intermediate filament proteins that are components of the nuclear lamina, a network which lies beneath the inner nuclear membrane. Lamins are the major structural proteins of the metazoan nucleus and play essential roles in the maintenance of nuclear integrity, organization of chromatin and gene regulation, as well as in organization of nuclear functions such as DNA replication and transcription. The lamins have been categorized into two groups, A-type and B-type lamins, based on expression patterns and biochemical properties. The B-type lamins are represented by lamins B1 and B2 that are encoded by the *LMNB1* and *LMNB2* genes respectively, as well as germ-cell specific B3 which is a splice variant of the *LMNB2* gene. All somatic cells types express at least one of the B-type lamins during development. The A-type lamins are encoded by a single gene *LMNA*, which gives rise to alternatively spliced lamins A, C and C2. The major A-type lamin isoforms lamin A and C (hereafter referred to as lamin A/C) are expressed in most differentiated somatic cells in a developmentally regulated manner while lamin C2 is restricted to germ cells. Mutations in human *LMNA* have been linked to a spectrum of degenerative genetic diseases that are termed as laminopathies. Most laminopathies arise due to a single point mutation in one of the *LMNA* alleles and are thus autosomal dominant. The majority of mutations affect striated muscles causing Emery-Dreifuss muscular dystrophy (EMD), limb-girdle muscular dystrophy or dilated cardiomyopathy, while other mutations are associated with progerias or lipodystrophies such as familial partial lipodystrophy (FPLD) or a peripheral neuropathy termed Charcot-Marie-Tooth disorder type 2B [1]–[7].

The homozygous lamin A/C knock-out mouse shows postnatal lethality with EMD-like symptoms and cardiomyopathy, and is considered to be a useful disease model [8], [9]. Various lamin A/C knock-in or transgenic mouse models have also been derived which express laminopathic mutations causing muscular dystrophy, cardiomyopathy or progeria, and these models mostly resemble their human disease counterparts [10], [11]. Heterozygous lamin A/C knock-out mice develop a late-onset cardiomyopathy, and show signs of cardiac dysfunction by 4 weeks of age but not in neonatal mice, suggesting a normal developmental program [12]. A possible explanation for this milder phenotype has been provided by the recent finding that the lamin A/C knock-out mouse line expresses a truncated lamin of size 54 kD, which is likely to be hypoactive [13]. In cell culture models, on the other hand, the expression of lamin mutations or reduction of lamin A/C expression causes widespread impairment of global gene transcription and gene regulatory pathways, including muscle and adipocyte differentiation pathways [1]–[7], [14], [15]. Moreover, truncating mutations in human lamin A leading to absence of lamin A can have strongly deleterious effects on fetal development [1]–[5]. Thus it is important to understand the role of lamin A/C level in cellular differentiation during embryonic development. In the present study, we have sought to determine the full differentiation potential of cultured embryonic stem (ES) cells with reduced levels of lamin A/C, which has not been described so far. Since the original disruption removed exons 8–11 [8], we attempted a different targeting strategy by disrupting the lamin A/C gene in exon 2 itself, so that a stable truncated protein was unlikely to be formed.

Mouse ES cells are derived from the inner cell mass of the early blastocyst stage of the embryo and retain their ability of self renewal and pluripotency *in vitro* and can form all three germ layers of the embryo proper during differentiation in culture [16]–[18]. The differentiation of ES cells in culture mimics the different stages of early embryonic development and provides information on their differentiation potential into different tissue types, which is not easily amenable to study in an animal model. Earlier studies have shown that mouse ES cells and embryonic carcinoma cells display either little or no lamin A/C expression in their undifferentiated state, whereas expression of lamin A/C is induced when these cells are differentiated in culture [19]–[22].

To ascertain the importance of lamin A/C in the process of early embryonic development and the lineage commitment of ES cells, we studied the effect of haploinsufficiency of lamin A/C in mouse ES cells. We assessed the extent to which reduction of A-type lamins affected the differentiation potential of heterozygous *Lmna* knockout ES cells into a broad range of cell types by analysis of lineage-specific markers in embryoid bodies (EBs) derived from these cells by *in vitro* culture. Our data show a marked preference for heterozygous *Lmna^+/−^* cells to form the visceral endodermal lineage, together with depletion of neuronal and myogenic lineages, indicating a skewed lineage differentiation program in lamin A/C haploinsufficient ES cells. Expression of lineage-specific markers showed similar trends in clones of mouse ES cells stably expressing shRNA against lamin A/C. Thus, our study shows that the relative level of lamin A/C expression is one of the critical determinants of the complex tissue-specific differentiation program.

## Results

### Lamin A/C haploinsufficiency does not affect pluripotency of mouse ES cells

**Figure 1:**
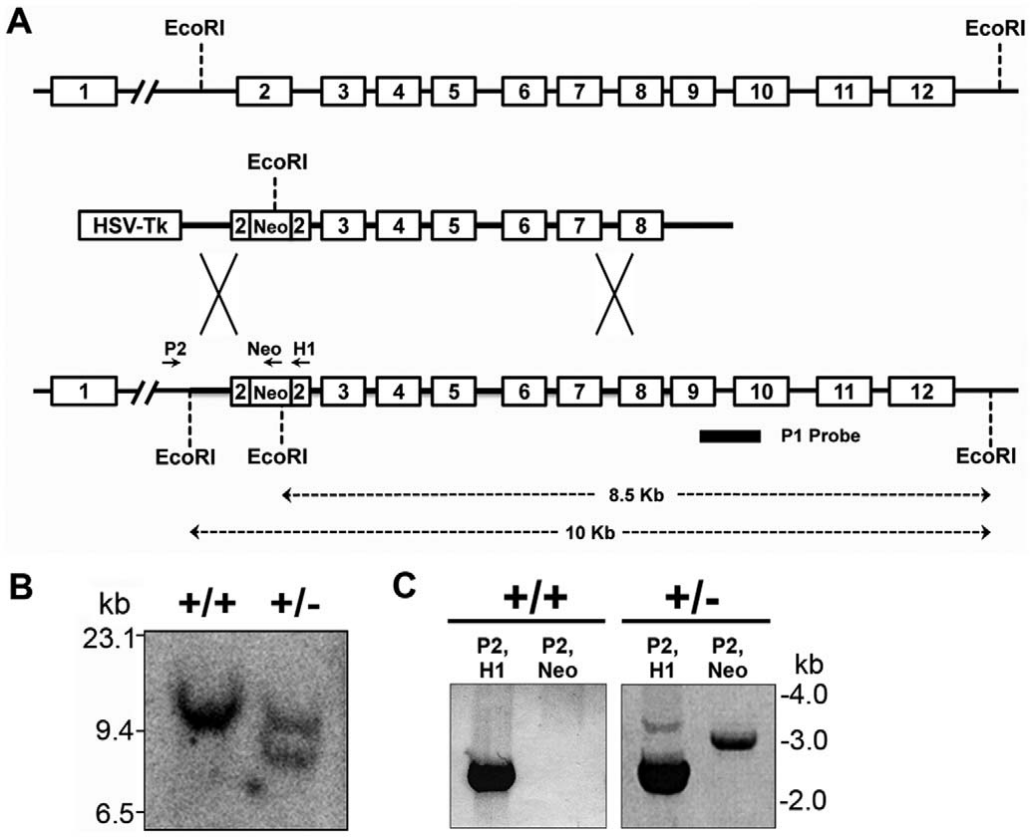
Targeted disruption of *Lmna* gene. (**A**) Schematic of *Lmna* genomic locus, targeting vector and the disrupted allele. The targeting vector had an insertion of a Neo^R^ cassette in reverse orientation in exon 2 for positive selection, and the HSV-TK gene at the 5′ end for negative selection. Positions of probe P1 and primers P2, Neo and H1 are indicated on the targeted allele. (**B**) Southern analysis of *Lmna* locus in wild-type (+/+) and *Lmna^+/−^* ES cells with P1 probe. (**C**) PCR analysis of *Lmna* locus in wild-type and *Lmna^+/−^* ES cells with the indicated primers.

In order to obtain an ES cell model of reduced lamin A/C expression, we decided to disrupt a single *Lmna* allele in mouse ES cells using the targeting strategy depicted in Fig. 1A. A targeting vector carrying the mouse *Lmna* genomic locus from the end of intron 1 to the start of exon 9, which was disrupted by insertion of a neomycin cassette in exon 2, and bearing the thymidine kinase gene was designed and used to target R1 ES cells. The targeted cells were selected in neomycin and gancyclovir for positive and negative selection, and screened for homologous recombination by Southern blot hybridization and also validated by PCR (Fig. 1B). The heterozygous targeted clone was expected to show an 8.5 kb band in addition to the 10 kb band seen in wild-type DNA after digestion with EcoRI followed by Southern blot hybridization with a probe spanning exons 9 and 10. The targeted clone was expected to show PCR amplification products of 3 kb (with P2+Neo) and 2.5 and 3.6 kb (with P2+H1) since it harboured the neomycin gene, whereas the wild-type allele should show only a 2.5 kb product with P2+H1. One clone was obtained that was confirmed to have a single targeted allele and was studied in detail.

**Figure 2:**
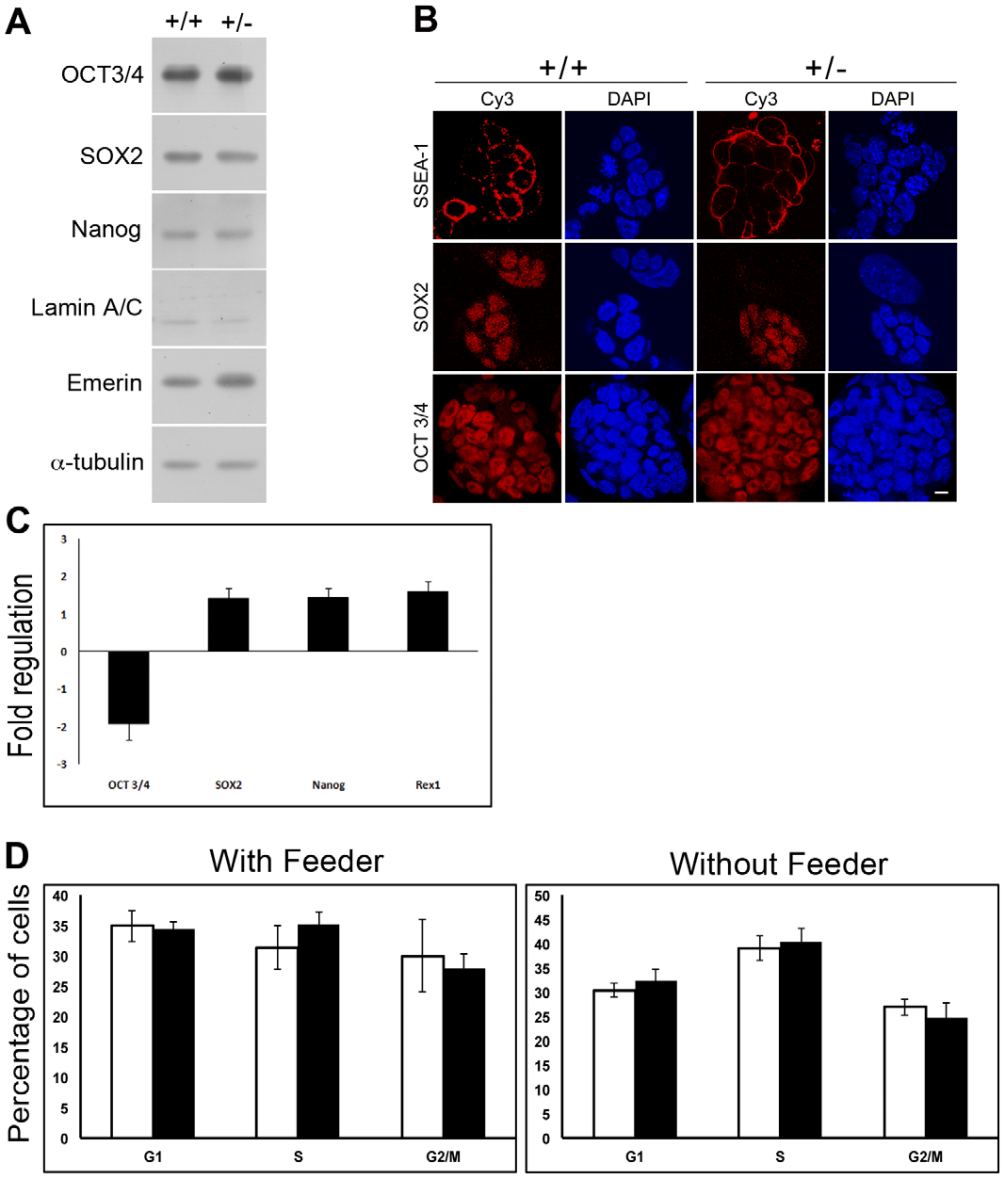
Analysis of pluripotency markers in lamin A/C haploinsufficient ES cells. (**A**) Immunoblot analysis of wild-type and *Lmna^+/−^* ES cells with antibodies to pluripotency markers Oct3/4, Sox2 and Nanog, as well as lamin A/C and emerin. α-tubulin was used as the loading control. Data is representative of two independent experiments. (**B**) Immunofluorescence analysis of pluripotency markers SSEA1, Sox2 and Oct3/4 in wild-type and *Lmna^+/−^* ES cells. The cells were counterstained with DAPI. Bar, 10 µm. (**C**) Quantitative RT-PCR analysis of transcripts for the indicated pluripotency markers in *Lmna^+/−^* ES cells. The fold-change values were normalized to wild-type ES cells. (**D**) Flow cytometric analysis of wild-type and *Lmna^+/−^* ES cells in presence (left panel) and absence (right panel) of MEF feeders. Percentages of cells in different phases of the cell cycle are indicated. Values represent mean±SD of three independent experiments.

The heterozygous *Lmna^+/−^* clone was initially screened for expression of well established stemness and pluripotency markers (Fig. 2). In mouse ES cells, a cohort of three transcription factors, the POU homeodomain protein, Oct3/4, the SRY-related HMG transcription factor, Sox2 and Nanog collaborate to regulate expression of genes essential for maintenance of pluripotency and self-renewal as well as repression of differentiation [23], [24]. Both the untargeted and heterozygous ES cells showed no significant differences in the expression of Oct3/4, Sox2 or Nanog proteins by immunoblot and immunofluorescence analysis, and no significant differences in transcript levels under undifferentiated conditions (Fig. 2A–C). Transcripts encoding Rex1, a pluripotency marker expressed in the inner cell mass cells of the blastocyst [25], also showed no significant difference in the *Lmna^+/−^* clone compared to wild-type ES cells (Fig. 2C). Lamin A/C levels were very low in both cell types, as expected. The levels of the inner nuclear membrane protein emerin or heterochromatin protein 1 isoforms (data not shown) were also not altered. Additionally, expression and localization of the stage-specific embryonic antigen (SSEA1) glycolipids at the cell surface were also identical in both the cell types. The panels in Fig. 2B also indicate that the colonies formed by both cell types were phenotypically indistinguishable and there was no obvious abnormality in nuclear morphology. Next, we carried out a cell cycle analysis of the cells grown in the presence or absence of feeder layers. The FACS analysis revealed no significant changes in the percentage of cells in the different phases of the cell cycle between the wild-type and the *Lmna^+/−^* clone (Fig. 2D). Taken together, these data suggest that the stemness and pluripotency potential of both the cell types are similar and the proliferative potential of the heterozygous lamin A/C knockout clone is not perturbed.

### Downregulation of lamin A/C in the heterozygous ES cells

**Figure 3:**
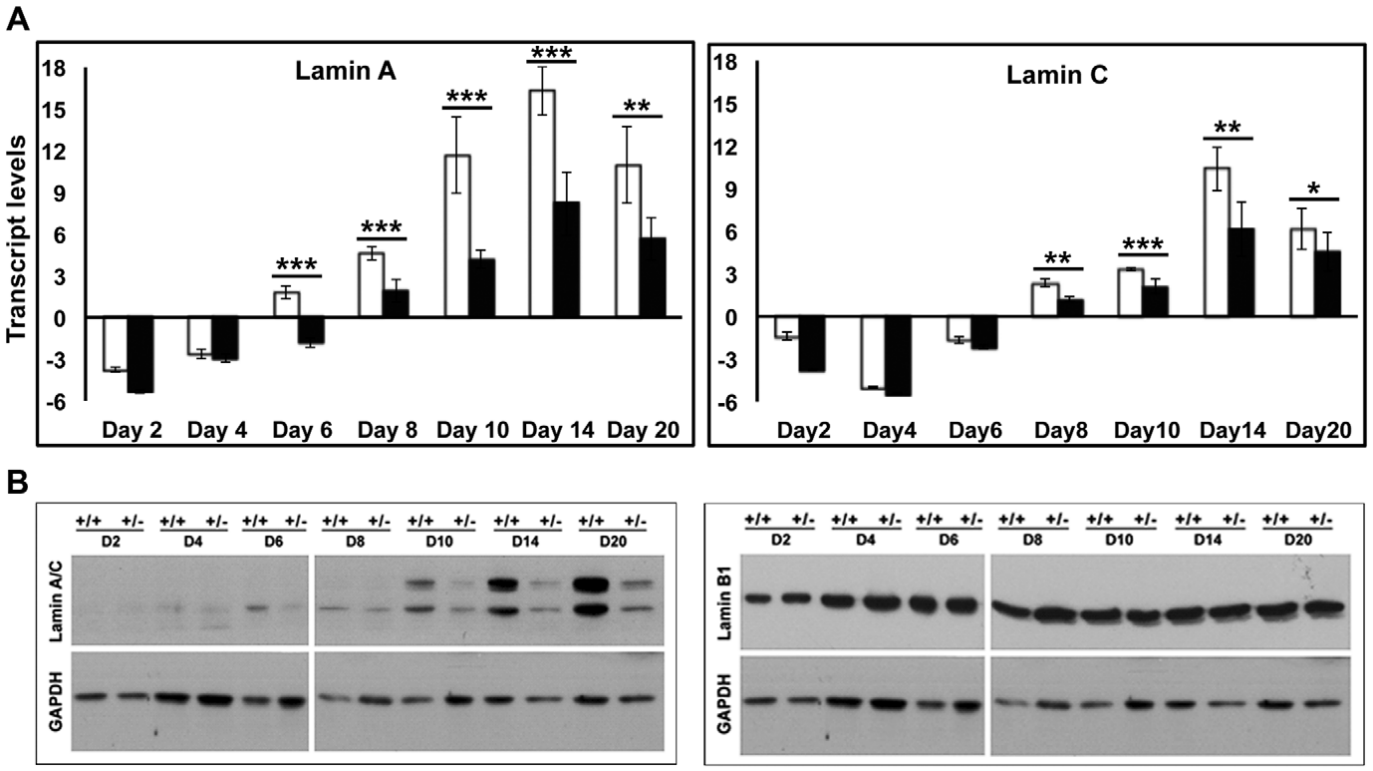
Analysis of lamin A/C expression in lamin A/C haploinsufficient cells. (**A**) Quantitative RT-PCR analysis of transcripts for lamin A (left panel) and lamin C (right panel) in differentiating EBs (day 2–20) of wild-type (open bars) and *Lmna^+/−^* cells (solid black bars), normalized to GAPDH. (**B**) Immunoblot analysis of expression of lamin A/C and lamin B1 during the differentiation of wild-type and *Lmna^+/−^* cells. GAPDH was used as loading control. * P≤0.05, ** P≤0.01, *** P≤0.001.

Since lamin A/C levels were very low in undifferentiated cells, we checked for differential expression of lamin A/C between wild-type and *Lmna^+/−^* cells in undifferentiated stem cells and cells spontaneously differentiated to EBs. The ES cells were initially made feeder-free by growing them in the absence of MEFs, and then differentiated by the hanging drop method. EBs were collected at days 0, 2, 4, 6, 8, 10, 14 and 20 and used for further experiments. Low levels of lamin A/C expression were observed at day 0 of differentiation in both wild-type and heterozygous cells, suggesting that a few peripheral cells had differentiated and hence expressed lamin A/C, as reported in previous studies [22]. Upon differentiation of ES cells, a gradual progressive increase in expression of lamin A/C was seen from day 4 onwards, which was at least 2-fold lower in the *Lmna^+/−^* cells compared to the wild-type, as indicated by both quantitative RT-PCR and immunoblot analysis (Fig. 3A,B). Interestingly, in undifferentiated cells and during the early days of differentiation higher amounts of lamin C protein were detected compared to lamin A in both cell types though both were transcribed. We found no evidence of any truncated lamin A/C protein product in *Lmna^+/−^* cells upon differentiation (data not shown).The expression levels of lamin B1 transcripts and protein showed no significant changes between the wild-type and *Lmna^+/−^* clone, suggesting the absence of a compensatory increase in lamin B1 to counter the reduction in A-type lamins. However, we were unable to detect transcripts encoding lamin B2 in the wild-type or *Lmna^+/−^* clone. This is consistent with a previous observation of lack of lamin B2 mRNA expression during ES cell differentiation, where the authors have proposed that either a splice variant of lamin B2 other than lamin B3 was present in ES cells or lamin B2 mRNAs were short-lived though the protein was stably expressed [22].

### Lamin A/C haploinsufficiency promotes visceral endoderm formation

ES cells are derived from the inner cell mass of an early blastocyst stage embryo, and maintain pluripotency in *in vitro* culture in the presence of LIF, an interleukin 6 class cytokine [26]. They maintain the ability to differentiate into all the lineages of the embryo proper but lack the ability to form trophectoderm under normal conditions of differentiation [27]. Differentiation of ES cells *in vitro* by aggregation of cells to form EBs is known to recapitulate the initial steps of embryonic development [28], [29]. The EBs form an outer layer of polarized primitive endoderm surrounding an inner mass of nonpolarized cells known as primitive ectoderm. The two layers are separated by a layer of basal lamina secreted by the primitive endoderm. As EB development approaches gastrulation, the primitive ectoderm undergoes a process of epithelialization and cavitation similar to the preimplantation stage embryo. During gastrulation this primitive ectoderm in EBs gives rise to the primitive streak and subsequently to all the three germ layers of the embryo proper. Prior to gastrulation the primitive endoderm is known to give rise to the visceral endoderm. Thus *in vitro* differentiation of EBs has been well documented to mimic the initial stages of embryogenesis [30].

**Figure 4:**
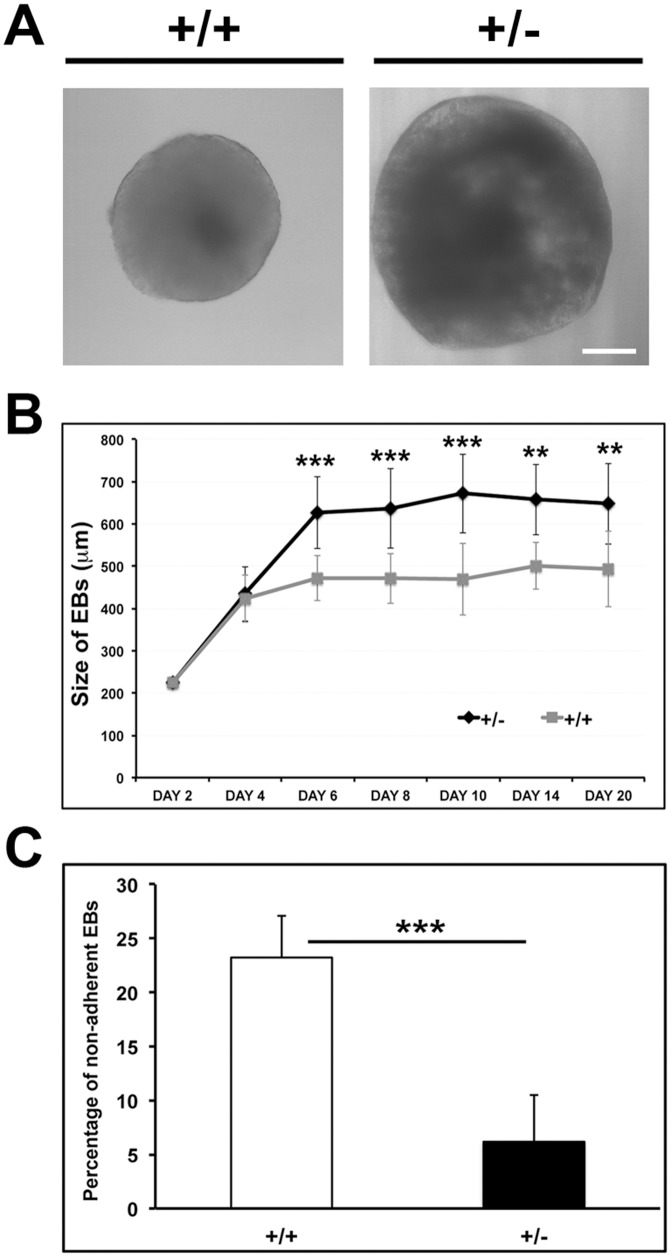
Comparison of sizes of EBs during differentiation. (**A**) Bright field images of representative EBs at day 8. Bar, 100 µm. (**B**) The diameter of EBs was measured under an inverted microscope and quantitative analysis of mean±SD from three independent experiments is shown (n = 50 EBs). (**C**) The adherence capacity of EBs on gelatinized petri dishes was estimated by counting the number of non-adherent EBs on day 6 of differentiation and plotting as a percentage of total EBs. Values represent mean±SD of three independent experiments. ** P≤0.01, *** P≤0.001.

**Figure 5:**
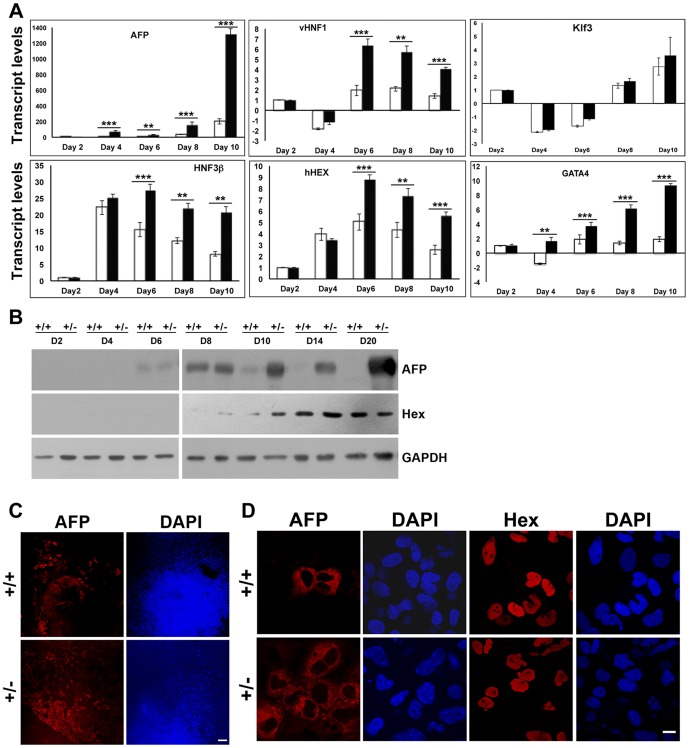
Analysis of visceral endodermal markers in lamin A/C haploinsufficient EBs. (**A**) Quantitative RT-PCR analysis of transcripts for the indicated visceral endodermal markers from day 2 to day 10 in wild-type (open bars) and *Lmna^+/−^* EBs (solid black bars), normalized to GAPDH. (**B**) Immunoblot analysis of expression of AFP and Hex during the differentiation of wild-type and *Lmna^+/−^* EBs from day 2 to day 20. Data is representative of two independent experiments. (**C**) Immunofluorescence analysis of AFP expression in whole EBs at day 10. Bar, 100 µm. (**D**) Immunofluorescence analysis of AFP and Hex expression in EBs at higher magnification. Bar, 10 µm. ** P≤0.01, *** P≤0.001.

We have compared lineage specification events in spontaneously differentiating wild-type and lamin A/C haploinsufficient EBs by marker analysis upto day 20, when most terminally differentiated cells have appeared. In most experiments, expression of regulatory factors and early markers has been analyzed by quantitative RT-PCR analysis upto day 10 of differentiation, while expression of late differentiation markers, which are generally more abundant, has been determined by immunoblot and immunofluorescence assays at later stages. During the course of differentiation, the *Lmna^+/−^* EBs were found to be larger in size and have greater adherence compared to day-matched wild-type EBs (Fig. 4), and also formed a higher number of cystic EBs (data not shown). One plausible reason for this anomaly could be increased visceral endoderm formation. To address this issue, we studied the expression of α-fetoprotein (AFP), a marker of visceral endoderm [31], in the differentiating EBs. Interestingly, transcript levels of AFP were higher in *Lmna^+/−^* EBs from day 4 onwards and a substantial upregulation was observed after day 8 in both transcript and protein levels (Fig. 5A,B). To ascertain whether the increase was due to higher number of cells of visceral endodermal lineage, the EBs corresponding to day 10 of differentiation were stained with anti-AFP antibody. The data revealed an increase in the number of cells staining positive for AFP in the *Lmna^+/−^* EBs compared to wild-type EBs (Fig. 5C). We noted that AFP localization was similar in both types of cells and nuclear morphology of lamin A/C haploinsufficient cells was not altered.

Expression of the AFP gene is regulated by several factors such as Klf3/BKLF, an early regulator of AFP transcription, and variant hepatic nuclear factor 1 (vHNF1), a potent transcription factor governing visceral endoderm formation [32]. Although we did not observe a significant change in Klf3 transcript levels, vHNF1 transcripts showed more than 2-fold increase in the *Lmna^+/−^* EBs from day 6 (Fig. 5A). Various *in vitro* and *in vivo* studies have shown that the zinc finger transcription factors Gata4 and Gata6 function upstream of HNFs and, together with vHNF1 are required for visceral endoderm differentiation [33]–[35]. While Gata6 transcripts showed no change in expression (data not shown), an increase in Gata4 expression was detected in the *Lmna^+/−^* EBs from day 4 onwards (Fig. 5A). HNF3β/FoxA2 is a forkhead transcription factor which is initially expressed in the visceral endoderm where it is required for normal primitive streak morphogenesis [36] and subsequently in the primitive streak during mesendoderm formation and later in nascent endoderm [37]. FoxA2 showed a significant 2-fold increase in transcript levels in the heterozygous EBs (Fig. 5A). It was not possible to detect vHNF1, Gata4 or FoxA2 protein expression in EBs, probably due to their low abundance. Hex, a regulatory target of FoxA2 that regulates the formation of various organs from the foregut [38], showed nearly 2-fold increase at the transcript level in the heterozygous EBs, and a small increase in amounts of protein at day 8–10 (Fig. 5A,B). The above results imply that differentiation to the visceral endodermal lineage is strongly promoted in the background of lamin A/C haploinsufficiency.

### Lamin A/C haploinsufficiency impairs neuronal differentiation

**Figure 6:**
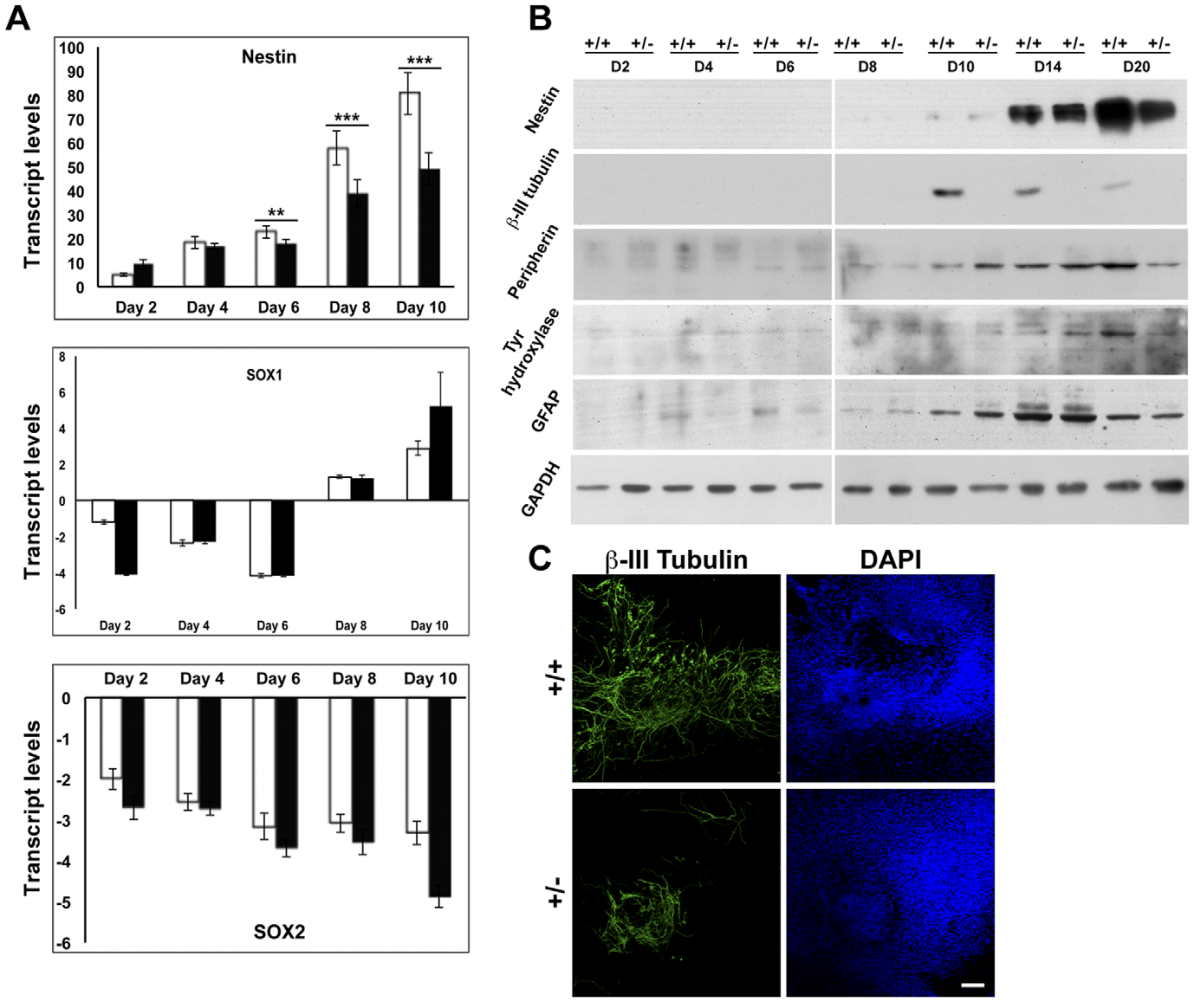
Analysis of ectodermal markers in lamin A/C haploinsufficient EBs. (**A**) Quantitative RT-PCR analysis of transcripts for the indicated neuronal markers from day 2 to day 10 in wild-type (open bars) and *Lmna^+/−^* EBs (solid black bars), normalized to GAPDH. (**B**) Immunoblot analysis of expression of ectodermal markers during the differentiation of wild-type and *Lmna^+/−^* EBs from day 2 to day 20. Data is representative of two independent experiments. (**C**) Immunofluorescence analysis of β-III tubulin expression in whole EBs at day 12. Bar, 100 µm. ** P≤0.01, *** P≤0.001.

Neuronal differentiation can be induced in ES cells by addition of exogenous factors like retinoic acid and sonic hedgehog or by spontaneous differentiation of EBs without the addition of exogenous factors, since EBs have the potential to give rise to neuroectoderm and related lineages. We have studied the effects of lamin A/C haploinsufficiency on neuronal development in spontaneously differentiated EBs by analysis of well documented neural markers [39]. The neural progenitors express a type VI intermediate filament protein called nestin that gets replaced upon differentiation by cell-type specific intermediate filament proteins. The expression of nestin transcripts was reduced in *Lmna^+/−^* EBs by day 8 of differentiation, which was evident at the protein level by day 20 of differentiation (Fig. 6A and B). Neural progenitor cells (NPCs) differentiate to form neurons and glia, which represent a variety of support cells for neuronal development and function. Sox1 and Sox2, transcription factors of the SoxB1 family, are expressed in proliferating NPCs and regulate their self-renewal and differentiation [40], [41]. The transcripts for both these genes did not show a significant difference in wild-type and *Lmna^+/−^* EBs by day 10, suggesting that there might not be a direct effect on the determination of NPC fate by this stage (Fig. 6A). On the other hand, we detected a marked reduction in the expression of β-III tubulin, a pan neuronal marker from day 10 onwards in the EBs with lamin A/C haploinsufficiency by immunoblot analysis as well as immunofluorescence assays (Fig. 6B,C). Furthermore, peripherin, a type III intermediate filament protein present in peripheral neurons, and tyrosine hydroxylase, a marker for dopaminergic neurons, also showed decrease in expression in *Lmna^+/−^* EBs by day 20 of differentiation (Fig. 6B). However, glial acid fibrillar protein (GFAP), a type III intermediate filament protein which is a specific marker for astrocytes, showed no significant change between the wild-type and *Lmna^+/−^* EBs. Olig1 and Olig2 transcription factors that are involved in oligogenesis [42] were undetectable in both types of cells upon differentiation (data not shown), probably due to their low abundance in spontaneously differentiated EBs. These results suggest a decreased commitment of lamin A/C haploinsufficient cells into the neuronal lineage, with no detectable effects on glial differentiation.

### Lamin A/C haploinsufficiency downregulates myogenic differentiation

**Figure 7:**
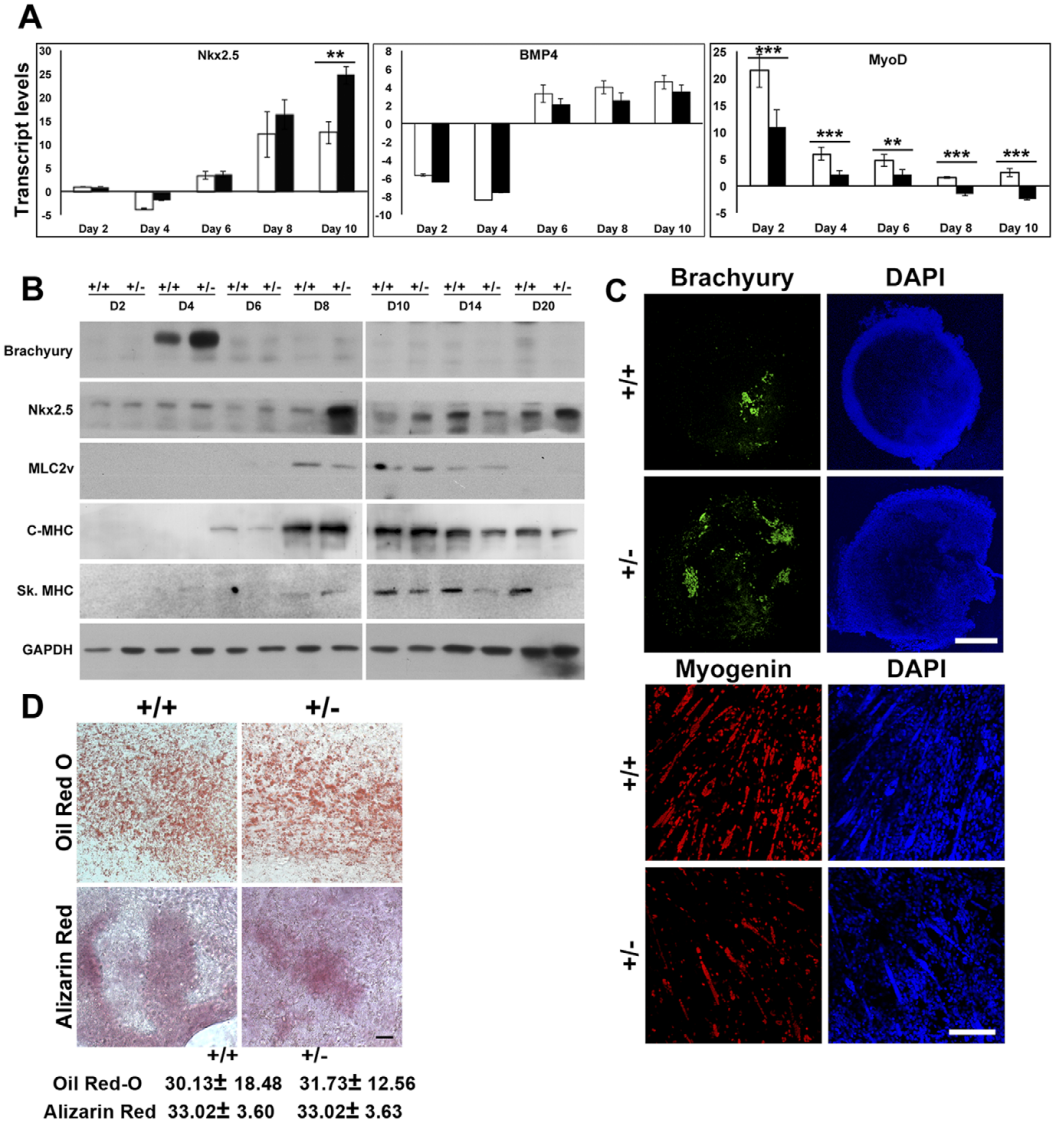
Analysis of mesodermal markers in lamin A/C haploinsufficient EBs. (**A**) Quantitative RT-PCR analysis of transcripts for the indicated mesodermal markers from day 2 to day 10 in wild-type (open bars) and *Lmna^+/−^* EBs (solid black bars), normalized to GAPDH. (**B**) Immunoblot analysis of expression of mesodermal markers during the differentiation of wild-type and *Lmna^+/−^* EBs from day 2 to day 20. Data is representative of two independent experiments. (**C**) Immunofluorescence analysis of brachyury (day 4) and myogenin (day 20) expression in whole EBs. Bar, 100 µm. (**D**) Oil red-O and Alizarin red staining of wild-type and *Lmna^+/−^* EBs on day 20 of differentiation. Bar, 100 µm. Values represent mean±SD of three independent experiments for quantitation of staining. ** P≤0.01, *** P≤0.001.

Earlier studies have established that *Lmna* homozygous knock-out mice show EMD-like symptoms, with severe dystrophy of skeletal muscles and cardiac atrophy, and die by 6–8 weeks of age [8], whereas heterozygous knockout mice exhibit cardiac abnormalities in the adult [12]. To determine whether reduction of lamin A/C affects the commitment and differentiation of mesodermal lineage during embryogenesis, we studied the expression pattern of various lineage specific markers during the course of differentiation. Brachyury, a T-box transcription factor, is the earliest known marker of mesodermal lineage commitment and is expressed in the mesendodermal population and later in the nascent mesoderm population [43], [44]. We compared the expression levels of brachyury in wild-type and *Lmna^+/−^* EBs and observed that in both of these cell types brachyury followed a characteristic transient expression pattern which peaked at day 4, corresponding to the gastrulation stage; however, *Lmna^+/−^* EBs had approximately 2-fold higher levels of brachyury protein expression compared to the wild-type (Fig. 7B). In immunofluorescence assays of EBs at day 4, a higher fraction of cells stained positive for brachyury in *Lmna^+/−^* EBs in comparison with wild-type (Fig. 7C).

During development, the mesodermal population gives rise to various lineages consisting of skeletal muscle, cardiac, adipocyte or osteocyte cell types. To examine the effects of lamin haploinsufficiency on myogenesis, we analyzed the expression of known muscle markers [45], [46]. MyoD transcripts were significantly reduced in *Lmna^+/−^* EBs compared to EBs derived from wild-type ES cells (Fig. 7A). Immunoblot analysis with skeletal-specific MHC antibody clearly showed reduced levels in *Lmna^+/−^* EBs at later stages of differentiation (Fig. 7B). Furthermore, the number of myotubes stained by myogenin antibody showed a significant decrease in *Lmna^+/−^* EBs by day 14 (Fig. 7C). Therefore, in accordance with previous reports, our results implicate lamin A/C in ensuring proper differentiation of myocytes to myotubes.

Nkx2.5 is a transcription factor expressed in precursors of the cardiac mesoderm, where it interacts with Gata4 [47], and is involved in the earliest stages of mesodermal commitment to cardiomyocytes but does not affect other pathways of mesodermal differentiation. Immunoblot and quantitative RT-PCR analysis showed that there was no significant change in Nkx2.5 expression in *Lmna^+/−^* EBs as compared to wild-type at transcript or protein levels, except for an increase in transcripts at day 10 (Fig. 7A,B). Expression of cardiac-specific MHC or MLC2v was also not significantly altered in *Lmna^+/−^* EBs (Fig. 7B). Bmp4, a transcription factor involved in mesoderm lineage commitment and heart and bone formation showed no significant change in transcript levels between wild type and haploinsufficient ES cells [48]. Gata5, another transcription factor involved in cardiogenesis together with Gata6 and Gata4 and expressed predominantly in endocardial cells [49], did not show any change at the transcript level (data not shown). We also estimated the adipogenic potential of *Lmna^+/−^* EBs by Oil red-O staining of lipid in oil droplets formed in adipocytes, but no significant changes were observed between the two cell types by day 20 (Fig. 7D). Osteogenic potential was analyzed by staining with Alizarin red, a dye used to stain Ca^++^ ions in the extracellular matrix but no significant differences in staining were observed by day 20 (Fig. 7D). Hence haploinsufficiency of lamin A/C has a profound effect on the process of myogenesis, but no discernible effects on cardiac, adipocyte or osteocyte lineage specification during early mesodermal differentiation.

### Lamin A/C knock-down cells simulate differentiation potential of haploinsufficient cells

**Figure 8:**
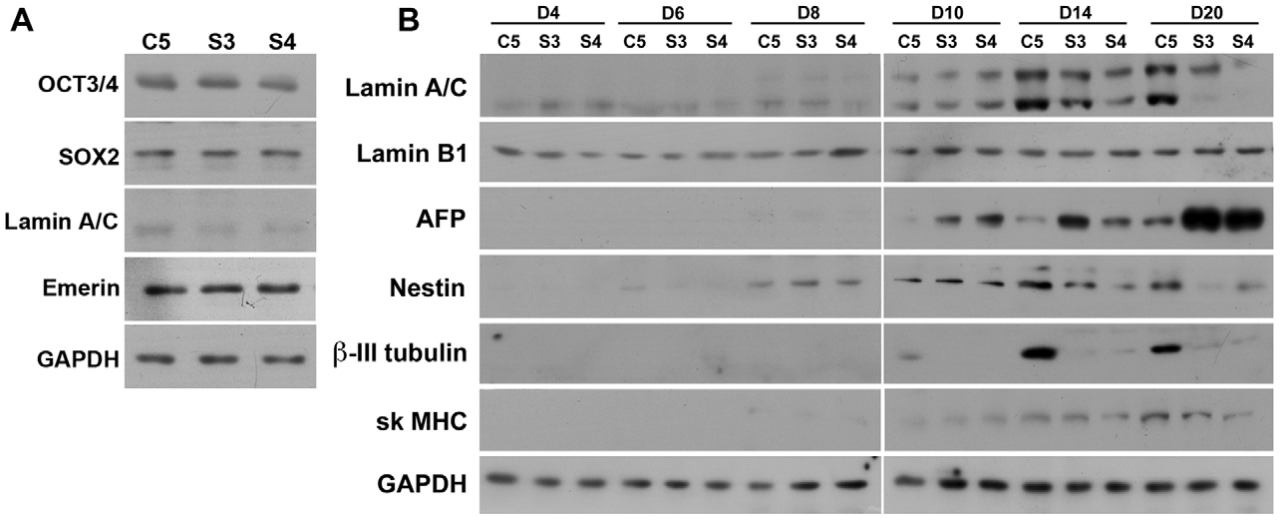
Analysis of pluripotency and lineage markers in lamin A/C knock-down ES clones. (**A**) Immunoblot analysis of control shRNA clone (C5), and lamin A/C shRNA clones (S3 and S4) with antibodies to pluripotency markers Oct3/4 and Sox2, as well as lamin A/C and emerin. GAPDH was used as the loading control. (**B**) Immunoblot analysis of expression of lamin A/C, lamin B1, AFP, nestin, β-III tubulin and skeletal muscle MHC during the differentiation of C5, S3 and S4 EBs from day 4 to day 20. GAPDH was used as the loading control. Data is representative of two independent experiments.

Stable mouse ES cell lines expressing shRNA against endogenous lamin A/C were derived in order to substantiate the results obtained with the lamin A/C haploinsufficiency clone. Two independent lamin A/C shRNA clones S3 and S4 showing 50–60% reduction in expression of lamin A/C at later days of differentiation and a control shRNA clone C5 were used. Initial studies with the undifferentiated cell lines showed similar expression of stemness markers Oct3/4 and Sox2, as well as lamin-binding protein emerin in lamin A/C shRNA and control shRNA cells (Fig. 8A). Low levels of lamin A/C expression were observed in undifferentiated cells as expected. The clones were differentiated by hanging drop method as described previously and EBs were collected at days 4,6,8,10,14 and 20. Notably, the EBs formed from shRNA clones S3 and S4 were larger in size than control C5 (data not shown) as seen in the case of *Lmna^+/−^* EBs. Embryoid bodies were examined for expression of various lineage specific markers by western blot analysis (Fig 8B). The endodermal marker protein AFP showed a marked increase in expression in both the stable lamin A/C shRNA clones during the time course. On the other hand, the ectodermal markers β-III tubulin and nestin showed a comparative decrease in their expression in the lamin A/C shRNA clones. A decrease in the mesodermal marker skeletal myosin heavy chain was evident by day 20. The overall effects of lamin A/C knock-down are consistent with those obtained with the haploinsufficiency clone and hence support the proposal that expression level of lamin A/C is a critical determinant of lineage commitment during early embryonic development.

## Discussion

In this study, we have examined the developmental potential of lamin A/C haploinsufficient ES cells by analyzing their spontaneous differentiation into EBs. The *Lmna^+/−^* cells retained pluripotency markers and proliferative properties. Upon differentiation into EBs, the *Lmna^+/−^* cells expressed approximately half the normal levels of lamins A and C. The differentiated *Lmna^+/−^* EBs showed increased levels of visceral endoderm markers but lower levels of neuronal and myogenic markers compared to wild-type EBs. Similar trends in expression of lineage-specific markers were observed in lamin A/C shRNA EBs.

### Lamin expression in ES cells

Several studies have established the ES cell as a suitable model to study embryogenesis as it recapitulates the process of gastrulation and can give rise to various lineages under appropriate conditions [16]–[18]. Hence, the differentiation of lamin A/C haploinsufficient ES cells in culture can provide access to regulation of key events during lineage specification in the background of reduced lamin A/C expression. Furthermore, ES cells provide a valuable tool to study the role of A-type lamins in initial stages of embryogenesis since lamin A/C is a maternally inherited protein that exists in the oocyte and early stages of zygotic division, but lamin A/C is undetectable or present at very low levels in ES cells as these cells are derived from early blastocyst stage embryos [19], [20]. During development, lamin A/C reappears first in the trophectoderm and visceral endoderm and later in a spatio-temporal manner in various parts of the embryo proper [20], [21]. Expression of A-type lamins has been detected immediately before reduction of Oct 3/4 levels in stem cells as they start to differentiate [22]. Early embryonic studies with various animal models (murine, bovine, porcine) have mapped the time of disappearance and reappearance of lamin A/C protein to various stages of embryogenesis, suggesting that the event may depend upon the maternal to zygotic transition occurring at different stages of embryogenesis in these organisms[22], [50], [51].

Consistent with previous studies, we found very low expression of lamin A/C in ES cells. Upon spontaneous differentiation, the expression of lamins was evident by day 4 which corresponds to the timing of visceral endoderm formation. Interestingly, lamin C protein appeared a few days before lamin A during differentiation of ES cells under our conditions, for both normal and haploinsufficient cells, as well as shRNA clones, suggesting that an active mechanism might exist to downregulate lamin A expression during early differentiation. As the targeted allele in this study contains an insertion in exon 2 of *Lmna*, we expect reduction in levels of both lamins A and C, and this was observed from day 10 onwards. Although mice expressing only lamin C or only lamin A appear overtly normal, suggesting redundancy of function, these proteins are highly conserved and are likely to have unique functions which have not yet been identified [11]. A possibility suggested by our data is that lamin C, which lacks the –CAAX membrane anchorage motif, is preferred over lamin A in ES cells at early stages of differentiation when the nucleus is more plastic [52].

### Role of lamins in lineage specification

Visceral endoderm is one of the earliest known lineages in which lamin A/C is expressed [20]. We observed a substantial increase in expression of the visceral endodermal marker AFP in lamin A/C haploinsufficient or knock-down cells that paralleled the reduced expression of lamin A/C in these cells and was accompanied by upregulation of regulatory genes such as vHNF1 and Gata4. The temporal correlation between increased formation of visceral endoderm and reduced lamin expression in both haploinsufficiency and knock-down EBs suggests that normal lamin A/C levels are required for appropriate visceral endoderm formation. Since the visceral endoderm provides nutrients and various inducible signals for further lineage specification and anterio-posterio axis formation, its abnormal upregulation might have profound effects on differentiation pathways in surrounding cells. Hence the effects on lineage specification observed in this study could be a direct result of lamin A/C depletion or an indirect effect through its role on visceral endoderm mediated signaling or a combination of both.

Recent studies suggest that lamins play a crucial role in neural development. The B-type lamins have been shown to be functionally essential for neuronal migration and viability in the brain [11]. It has been recently reported that lamin C is the predominant A-type isoform that is expressed in astrocytes, oligodendrocytes, Purkinjee cells and neurons [53]. This variation in expression of the A-type lamins has been attributed to the presence of a brain specific microRNA, miR-9 that targets the 3′ UTR of prelamin A and not of lamin C. In another study, it has been shown that lamin A/C is complexed with numb interacting protein-1 (Nip1), a factor involved in the neuronal differentiation of stem cells [54]. The overexpression of Nip1 in P19 embryonal carcinoma cells led to upregulation of lamin A/C as well as genes involved in neuronal differentiation, while suppression of lamin A/C was shown to reduce Nip1 induced neuronal differentiation. We have observed reduction in nestin as well as neuronal lineage markers β-III tubulin, peripherin and tyroxine hydroxylase in EBs formed from lamin A/C haploinsufficient cells. Taken together, these results suggest that lamin A/C expression is closely linked to the neuronal differentiation program.

Since the majority of mutations in *LMNA* affect skeletal and cardiac muscle, the effects of laminopathic mutations or lamin A/C downregulation on differentiation of these tissues have been analyzed in several studies [9], [12], [55]–[57]. Although a direct role for lamins in muscle differentiation has not been reported, lamins have been proposed to undergo regulated rearrangements specifically during differentiation of normal myoblasts [58], [59]. Myoblast cell lines derived from *Lmna* knock-out mice showed reduced expression of certain muscle markers such as MyoD and pRb, upregulation of Myf5 and impaired differentiation; in heterozygous myoblasts, the delay in differentiation was intermediate between wild-type and knock-out cells [56]. Consistent with these observations, we also observed decreased expression of muscle markers and impaired myotube formation in *Lmna^+/−^* EBs during the differentiation time course. Though brachyury expression was increased in *Lmna^+/−^* EBs, we did not observe upregulation of differentiation of mesodermal lineages such as cardiac, skeletal muscle, osteocyte and adipocyte but we cannot rule out effects on other lineages like chondrocyte and blood islands which we have not examined. Increased brachyury expression might have contributed to higher levels of the endodermal markers FoxA2 and Hex in *Lmna^+/−^* EBs, a possibility suggested by the reported effects of brachyury induction on endodermal differentiation [44].Defects in cardiomyocytes derived from mice with lower levels of lamin A/C expression have been observed in mice older than 4 weeks of age, with no abnormality reported during development [9], [12], [60]. We also do not observe misregulation of the cardiac differentiation markers Nkx 2.5, cardiac MHC or MLC2v in lamin A/C haploinsufficient cells. Although muscle loss in lamin A/C knock-out mice is accompanied by bone loss and fat infiltration, with milder phenotypes seen in heterozygous knock-out mice [61], [62], we did not observe any differences in adipocyte or osteocyte differentiation in *Lmna^+/−^* EBs. In the recent report of a truncated A-type lamin in the lamin A/C knock-out mouse line [13], the authors have suggested that some of the documented features of this model may be attributed to hypoactivity or toxicity of the truncated product. Hence the data obtained from our lamin A/C haploinsufficient clones may not be directly comparable with the heterozygous knock-out mouse. Taken together, our results on mesodermal differentiation indicate that reduction of lamin A/C levels impairs myogenic differentiation but effects on cardiac, adipocyte or osteocyte differentiation are not evident at the early stages of EB differentiation analyzed in this study, thereby suggesting that lamin A/C levels may not be critical for early embryonic development of these tissues.

### Implications for laminopathies

Mutations in human *LMNA* cause a spectrum of >15 rare genetic diseases that affect different tissues such as skeletal muscle, cardiac muscle, adipose tissue, bone and peripheral neurons, and also cause premature ageing syndromes [1]–[7]. Laminopathies vary widely in their penetrance as well as age of onset (late/early). Furthermore, mutations that primarily affect one or two tissues occasionally show symptoms in other tissues also. Although patients showing absence of A-type lamins are rare, a family with autosomal dominant EMD has been reported with a heterozygous lamin A/C nonsense mutation (Q6X) [63], and a case of limb-girdle muscular dystrophy has been described with a heterozygous lamin A/C nonsense mutation (Y259X), both of which lead to lamin A/C haploinsufficiency. One of the homozygous offspring from the latter family showed fetal lethality with severe developmental defects, accompanied by abnormalities in nuclear morphology and total absence of lamin A/C [64].

Laminopathic cells exhibit widespread defects in nuclear morphology and structure which are often accompanied by defective nuclear mechanics, alterations in tissue-specific gene expression, modified protein-protein interactions, impaired response to DNA damage and proteasomal degradation of key regulatory proteins [1]–[7]. Signaling pathways that are altered in differentiated cells expressing laminopathic mutations or lowered levels of lamin A/C include mitogen-activated protein kinase (MAPK) signaling, retinoblastoma protein (pRb) dependent regulation, DNA repair, tumour growth factor-β (TGF-β)/Smad, Notch and NF-ĸB pathways, which can contribute to defective cell proliferation and differentiation, as well as cellular senescence. Our present findings indicate that lamin A/C depletion can also have profound effects on early lineage specification in ES cells, leading to aberrant differentiation of visceral endodermal, neuronal and myogenic lineages. These results have important implications for understanding the effects of lamin misexpression in a wide range of tissue types.

The differentiation of ES cells into EBs reflects embryonic differentiation potential as the microenvironment of cells in the EB harbours cells of different lineages as well as various extracellular matrix components, and permits cell-cell interactions. During early differentiation, ES cells give rise to a number of stem cell lineages, some of which contribute to adult stem cells later in life. Stem cells are involved in lineage specification, self-renewal, growth arrest and response to stress during development early in life and in tissue regeneration in the adult. Defective stem cell differentiation and proliferation early in development is likely to be lethal, whereas defects in adult stem cell proliferation and differentiation may lead to tissue degenerative diseases such as the laminopathies [65], [66]. Our data establish that EB differentiation can provide important insights into the role of lamin A/C in early differentiation events. Although we have studied the spontaneous differentiation of EBs, it is possible to enhance differentiation into distinct lineages by different protocols, thus allowing the study of specific cell types [16]–[18].

In conclusion, our study demonstrates that lamin A/C level is a critical determining factor in the differentiation of visceral endoderm, neuronal and myogenic lineages during early embryonic development. These findings provide support for the hypothesis that lamin A/C plays an important role in differentiation of specific lineages during development. Furthermore, our studies establish EB differentiation as a suitable model to study the functions of lamin A/C in differentiation.

## Materials and Methods

### ES cell culture and targeted disruption of lamin A/C gene

Undifferentiated mouse R1 ES cells [67] were routinely maintained on plasticware coated with mitomycin C-treated primary mouse embryonic fibroblast (MEF) feeders in ES-cell medium at 37°C in a humidified atmosphere containing 5% CO_2_. The ES-cell medium was composed of DMEM supplemented with 15% ES cell-qualified FBS (Hyclone), 1000 U/ml of leukemia inhibitory factor (LIF), 100 mM minimum essential amino acids, 2 mM GlutaMax (Gibco), 100 mM 2-mercaptoethanol, and 50 µg/ml of penicillin-streptomycin. Feeder-free ES cells were maintained in the same medium on plasticware coated with 0.1% gelatin.

A 7-kb fragment of mouse *Lmna* gene from the end of intron 1 to the beginning of exon 9 was isolated from a 129/Sv mouse genomic library. To construct the targeting vector, exon 2 was disrupted by insertion of a neomycin cassette in the reverse orientation (Fig. 1A). The entire sequence of the targeting construct was confirmed by automated DNA sequence analysis. The targeting vector was linearized with NotI and electroporated into R1 ES cells, followed by negative selection with gancyclovir at a final concentration of 2 µM for 4 days and positive selection with neomycin at a final concentration of 250 µg/ml for 10 days. The stable clones were picked, expanded, and screened for homologous recombinants by Southern hybridization of EcoRI-digested genomic DNA, using a probe (P1) spanning exons 9–10. One positive clone that was obtained was also validated by PCR amplification of the targeted genomic region with the primer pairs P2+H1 and P2+Neo listed in Table 1. The P2 sequence lies in intron 1 of *Lmna*, just upstream of the targeted region, whereas H1 and Neo sequences are present in the targeted segment.

**Table 1:** List of PCR primers.

	Gene	Forward primer 5′ to 3′	Reverse primer 5′ to 3′
1	Oct3/4	CTCGAACCACATCCTTCTCT	GGCGTTCTCTTTGGAAAGGTGTTC
2	Sox2	GCACATGAACGGCTGGAGCAACG	TGCTGCGAGTAGGACATGCTGTAGG
3	Nanog	CAGCCCTGATTCTTCTACCAGTCC	GGAGAGTTCTTGCATCTGCTGGAG
4	Rex1	TTGGGGCGAGCTCATTACTT	AGCTCTCCGTGAAGGCTTTG
5	Lamin A	TCTTCTGCCTCCAGTGTCACAG	CATGATGCTGCAGTTCTGGGAG
6	Lamin C	CCTTCGCACCGCTCTCATCAAC	GCGGCGGCTGCCACTCACAC
7	AFP	AGCCAGGCACTGTCCAAGCA	TGGCAGCACGTGGAGGCAAT
8	vHNF1	ATGCTCAGCGAGGACCCGTG	TGGGGGTGCCCTTGTTGAGG
9	hHex	TCACCAGCCTCGTGTCCTCCT	TCCGCGGGAACGGGTACAGA
10	HNF3β	CACCTGCCTCTGCGCTGAGT	TGGTGCTCGGGCTTCAGGTG
11	Klf3	GCGTCACCTGGCCTCAGCAT	GGATGCCTGGGCTCCGGATT
12	Gata4	AGTCCTGCACAGCCTGCCTG	TGTCCCGTCCCATCTCGCCT
13	Sox1	CCTTGCTAGAAGTTGCGGTC	TCACTCAGGGCTGAACTGTG
14	Nestin	ACTGTGGAATCACCAGGAGG	TGACCTTCTCCATCCTCCAC
15	Nkx2.5	CCAGAACCGTCGCTACAAGT	GGGTAGGCGTTGTAGCCATA
16	BMP4	CTGCGGGACTTCGAGGCGACACTTCT	TCTTCCTCCTCCTCCTCCCCAGACTG
17	MyoD	CGCTCGTGAGGATGAGCAT	AGCGTCTCGAAGGCCTCAT
18	GAPDH	ACCCAGAAGACTGTGGATGG	CACATTGGGGGTAGGAACAC
19	P2	ACCCAGCCCTGTGCATAACTCCT	-
20	H1	-	CCTCCAATGTGCGCTTCTCACT
21	Neo	-	ATACTTTCTCGGCAGGAGCA

### Generation of stable lamin A/C knock-down clones

Mouse ES cell clones stably expressing shRNAs were derived using the pcDNA6.2 GW/EmGFP-miR-LMNA or pcDNA 6.2 Gw/EmGFP-miR-negative control vectors (Invitrogen). The pcDNA6.2 GW/EmGFP-miR-LMNA vector was modified to express shRNA targeting mouse lamin A mRNA at position 1652–1673 (NM_001002011.2) by ligating oligonucleotides carrying the sense and antisense sequence between the BamHI and XhoI sites. The negative control vector expresses an shRNA sequence which is predicted not to target any sequence in the mammalian genome. The linearized shRNA vectors were electroporated into R1 ES cells, and selected for blasticidin resistance at a final concentration of 9 µg/ml as described earlier [68]. The stable clones were picked, expanded, and screened for lamin A/C knock down.

### Cell cycle analysis

ES cells were grown in the presence of MEF feeders or on gelatin-coated plates for 48 h. Cells were trypsinized, dissociated from feeders, fixed in cold 70% ethanol, treated with 10 µg/ml RNase A and stained with 50 µg/ml propidium iodide for 30 min at 37°C. Cell cycle analysis was performed on a FACS-Caliber flow cytometer (BD Biosciences) using Cellquest software. The analysis for each sample was done in triplicate and three independent biological replicates were performed for cells grown either in the presence or absence of feeders.

### ES cell differentiation

To induce spontaneous differentiation, feeder-free ES cells were suspended in ES-cell medium without LIF (differentiation medium). Cells were incubated (4.5×10^2^ cells per 30 µl) by the hanging drop method to form aggregates termed EBs as described by Metzger *et al.*, 1996 [69]. The EBs were maintained in suspension culture for 4 days (2 days as hanging drops and 2 days in bacteriological grade petridishes) in differentiation medium, and on the fifth day EBs were plated on tissue culture plates coated with 0.1% gelatin for attachment and spreading in culture till day 20 of differentiation. Differentiation media was changed after every 2 days of culture. For immunofluorescence assays, on day 5 the EBs were transferred onto 0.1% gelatin-coated glass coverslips placed in 6-well tissue culture plates.

### Antibodies and immunoblot analysis

Goat polyclonal antibodies to lamin A/C (N-18), Oct3/4, Nanog, peripherin, brachyury, GFAP and mouse mAb to tubulin and Gata4 were obtained from Santa Cruz Biotechnology. Mouse mAbs to emerin (clone 4G5) and SSEA1 were from Novocastra Laboratories and Chemicon, respectively. Rabbit polyclonal antibodies to GAPDH, lamin B1, Sox2, tyroxine hydroxylase, Hex, and MLC2v were obtained from Abcam. Mouse mAbs for nestin, C-MHC and Sk-MHC were obtained from Abcam and for β-III tubulin from R&D Systems. Rabbit polyclonal antibodies for AFP, Nkx2.5 and myogenin were procured from Santa Cruz Biotech. For immunoblot analysis, samples were lysed in Laemmli's sample buffer, boiled and electrophoresed through SDS-polyacrylamide gels. Gels were electroblotted onto PVDF membrane filters and blocked overnight in 5% BLOTTO in Tris-buffered saline containing 0.1% Tween-20. Filters were incubated with primary antibody for 2 h, followed by species-specific peroxidase conjugated-secondary antibody in Tris-buffered saline containing 0.1% Tween-20 for 1 h. Bound antibody was visualized using a chemiluminescence kit from Roche Applied Science Inc.

### Immunofluorescence microscopy

ES cells were fixed with 4% formaldehyde in phosphate-buffered saline (PBS) for 10 min followed by treatment with 0.5% (vol/vol) Triton X-100 for 6 min at room temperature. Cells were then incubated with 0.5% gelatin in PBS for 1 h followed by primary antibody for 1 h and then Cy3- or FITC-conjugated secondary antibody for 1 h at room temperature. EBs grown on coverslips were fixed in 4% paraformaldehyde for 30 min. After permeabilization and blocking as described above, EBs were incubated with primary antibody at 4°C overnight followed by secondary antibody for 1 h. Samples were mounted in Vectashield (Vector Laboratories) containing 1 µg/ml DAPI. Fluorescence microscopy was performed on an LSM510 META/NLO confocal microscope (Zeiss) with 63× and 10× objective lenses. EBs were imaged in the bright field mode using a 10× objective lens. Images were analyzed with LSM510 META software and assembled using Adobe Photoshop.

### Quantitative RT-PCR Analysis

Total RNA was extracted from ES cells at indicated time intervals and 1 µg of RNA was reverse transcribed using Superscript II reverse transcriptase kit (Invitrogen) as per the manufacturer's instructions. Amplification of PCR products was quantitated using SYBR green dye (ABI), and fluorescence was monitored on an ABI prism 7900 HT sequence detection system. Melting curve analysis was done for each amplicon. The 2^−ΔΔCt^ method was used for quantitation with glyceraldehyde 3-phosphate dehydrogenase (GAPDH) as endogenous control. The analysis for each gene was done in triplicate and three independent biological replicates were performed. The gene specific primers used for the analysis are given in Table 1.

### Oil red-O staining

Adipocyte differentiation was quantified by staining lipid droplets in differentiating or mature adipocytes with Oil red-O staining solution as described previously [70]. EBs at day 20 of differentiation were fixed with 4% paraformaldehyde, washed with distilled water and rinsed with 60% isopropanol. The EBs were stained with freshly prepared Oil red-O stain for 15 min, rinsed with 60% isopropanol and then with distilled water. After the images were taken on an Axioscope inverted microscope (Zeiss) with a 10× objective lens, the Oil red-O was extracted using isopropanol and the absorbance was determined at 510 nm. The number of cells was determined by a standard colorimetric assay using crystal violet. After elution, the cells were rinsed with water and then 0.5% of crystal violet in 20% methanol was added for 30 min. Any unbound dye was rinsed off with distilled water and the bound dye was eluted with 10% acetic acid and absorbance was determined at 590 nm. Oil red-O staining was normalized to the number of cells in each sample.

### Alizarin red staining

Osteogenesis potential of EBs at day 20 was quantified by staining of mineralized extracellular matrix with Alizarin red. EBs fixed with 4% paraformaldehyde were rinsed with distilled water and stained with 40 mM Alizarin red S for 20 min and washed with deionized water [71]. Alizarin red was extracted from the stained EBs with 10% acetic acid and measured at 405 nm. Alizarin red staining was normalized to extracted protein content per sample. The images were acquired using an Axioscope inverted microscope (Zeiss).

### Statistical analysis

Data presented are averages±standard deviation for three independent biological replicates. When comparing two data sets, P values were generated using Student's t-test. P≤0.05 was considered significant, P≤0.01 was considered very significant and P≤0.001 was considered extremely significant at 95% confidence level.
